# *Nigella sativa* Oil Improves Motor Skill Learning of Albino Mice: *In Vivo* and *In Silico* Investigations

**DOI:** 10.1155/2023/8498066

**Published:** 2023-08-25

**Authors:** Md. Siam Hossain, Abu Baker Seddique, Suraiya Sharmin, Md. Mamun Or Rashid, Arifin Islam, Md. Monir Hossain

**Affiliations:** ^1^Department of Pharmacy, Noakhali Science and Technology University, Sonapur, Noakhali 3814, Bangladesh; ^2^Department of Biochemistry and Molecular Biology, University of Rajshahi, Rajshahi 6205, Bangladesh; ^3^Department of Accounting & Information Systems, Jagannath University, 9-10, Chittaranjan Avenue, Dhaka 1100, Bangladesh; ^4^Department of Pharmacy, Jagannath University, 9-10, Chittaranjan Avenue, Dhaka 1100, Bangladesh

## Abstract

Experimental evidences demonstrated that *Nigella sativa* oil (NSO) can restore neuronal integrities and processes by increasing the neuronal density, decreasing apoptosis, preventing inflammatory processes, and improving the neurogenic cells in the hippocampus. This refurbishment enhances the learning process and memory. The antioxidant defense mechanism of NSO slows down the process of neurodegeneration and motor deficit. The present study aimed to investigate the effects of NSO on motor skill learning using the single pellet reaching task method on Swiss albino mice, followed by *in silico* studies. Mice (total of 16) were randomly divided into the control group and treatment group (*n* = 8). The treatment group received 1 ml/kg b.w. NSO orally once daily for 7 days, and a control group received 1 ml/kg normal saline instead of NSO in a similar manner. The average success rate due to ingestion of NSO in the treatment group mice increased significantly (*P* < 0.05) compared to controlled mice. Molecular docking analysis revealed that thymoquinone, carvacrol, thymohydroquinone, *p*-cymene, and *t*-anethole have binding affinities for the *α*-amino-3-hydroxy-5-methyl-4-isoxazolepropionic acid receptor (AMPA-R) that ranges from (-5.1 to -6.2) kcal/mol, which is comparable to the reference ligand glutamic acid binding affinity with AMPA-R (-6.6 kcal/mol). Thymoquinone and carvacrol formed hydrogen bonds with AMPA receptor at TYR61, SER142, and SER143 residues, comparable to the binding affinity of glutamic acid. ADMET analysis reported that all the compounds have higher bioavailability (>90%) and can cross the BBB easily (logBB> 0.3). Based on our experimental data and *in silico* report, we concluded that the enhanced motor skill learning effects of NSO are due to presence of potent antioxidants—thymoquinone and carvacrol—which might serve as AMPA receptor agonists. These phytoconstituents may play role in synaptic strengthening and promote experience-dependent motor skill learning.

## 1. Introduction

Throughout life, animals have a surprising ability to learn new motor skills. Developing new dendritic spines on pyramidal cells in the motor cortex is necessary for the acquisition of new abilities [1]. The motor cortex (M1), especially layers 2/3 and 5 (L2/3 and L5), which undergo remodeling during learning, is crucial for the development of motor abilities [2]. Motor learning enriches synaptic associations in M1 by coordinating the clustering of dendritic spines, constructing the structural basis for storing motor memory [3]. M1 is necessary for skilled voluntary movements because during these activities, M1 neurons vigorously discharge to encode several properties, including force, direction, and speed of spontaneous movements [4]. Activation of *α*-amino-3-hydroxy-5-methyl-4-isoxazolepropionic acid (AMPA) type glutamate receptors induces fast excitatory transmission, boosting memory and enhancing task-related behavior [5]. Moreover, increased postsynaptic AMPA receptors were reported in the early phase of motor learning [6]. Long-term potentiation (LTP) is a component of a continuum of types of neural adaptation, some leading to restorative alterations such as motor learning.

Previous studies have shown that cerebellar norepinephrine (NE) depletion or beta-adrenergic receptor blockade significantly impaired runway task performance [7]. Likewise, a strong correlation was observed between the loss of beta-adrenergic receptors and impaired performance in aged rats [8]. Diet that comprises high antioxidants could enhance performance on a motor learning task and reverse an age-induced decline in cerebellar beta-adrenergic receptor function [9]. Oxidative stress-induced cognitive performance was enhanced by antioxidant supplements [10]. The pathophysiologies of many neurodegenerative diseases, such as Alzheimer's and Parkinson's diseases, are expressed because of oxidative stress and reactive oxygen species [11]. Medicinal herbs have been shown in numerous studies to have strong antioxidant properties, making them suitable sources for treating various disorders brought on by oxidative stress [12, 13]. The reduction of free radical production can reduce oxidative stress [14, 15].

Medicinal plants have drawn significant attention in the current years because of their fewer toxic effects, availability, and lower price than synthetic substances, making them excellent and simple selections for the invention of new drugs used in neuronal diseases [16]. *Nigella sativa* Linn (*N. sativa*) is an annual flowering herb belonging to the family of Ranunculaceae [17]. It is well known for the ameliorative effects of neurological disorders, such as psychiatric dysfunction and memory loss [18]. Due to its potentiality to prevent the free radical damage, *N. sativa* has been utilized extensively to treat neurological illnesses like Parkinson's and Alzheimer's diseases [19]. Almost 35 active ingredients have been found in *N. sativa* including thymoquinone (28–45%), *α*-pinene, sesquiterpene, longifolene (1–8%), *t*-anethole (1–4%), carvacrol (6–12%), *p*-cymene (7–15%), dithymoquinone, thymohydroquinone, and 4-terpineol (2–7%). The primary active component of *N. sativa* seeds is thymoquinone (2-isopropyl-5-methyl-1,2-benzoquinone), which has a variety of pharmacological effects, including antibacterial, antifungal, anti-inflammatory, antioxidant, and anticonvulsant properties [20].

Black seed oil, usually referred to as *Nigella sativa* oil (hence, NSO), is a highly regarded medicinal solvent that has been used for centuries to treat a variety of illnesses. It demonstrated powerful antioxidant and neuroprotective properties [20]. Previous studies showed that NSO enhanced the survival of neurogenic cells in the hippocampus and reduced the functional loss of striatal dopaminergic neurons in Parkinson's bearing mice. It has been widely shown that NSO and its component thymoquinone can restore the health and functionality of neurons by boosting their density, lowering their rate of death, and halting inflammatory reactions. Thymoquinone shields cells from oxidative agents, preventing cell damage and neurodegeneration in the brain [21].

Molecular docking is a popular and primary strategy in drug development programs. It can predict the molecular interactions between a protein and a ligand in the bound state. Moreover, this technique indicates the information about the preferred orientation, affinity, and interaction of a ligand in the binding site of a protein [22]. In this investigation, we concentrated on the AMPA receptor, a glutamate-gated ion channel that is principally in charge of synaptic plasticity, including long-term potentiation (LTP) of the synaptic transmission, and mediates the majority of excitatory synaptic transmission in the brain [23]. LTP may facilitate motor learning through the neuronal changes [24].

To the best of our knowledge, no research was carried out to observe the effect of NSO on the acquisition of motor skills in mouse models. To address this, we employed the single pellet reaching task, a more accurate test of motor function because it closely resembles the motions and actions that animals usually engage in their surroundings. We also investigated the molecular docking of the important phytoconstituents present in NSO (ligands) by focusing on AMPA receptors (protein).

## 2. Materials and Methods

### 2.1. *In Vivo* Experimental Procedures

#### 2.1.1. Experimental Animals

Male Swiss albino mice (22–25 g), aged 6-7 weeks, were purchased from the animal house of the International Centre for Diarrhoeal Disease Research, Bangladesh (ICDDR, B). They were kept in plastic cages in the animal laboratory of the Dept. of Pharmacy, Noakhali Science and Technology University, for 7 days before the experimental session to adapt with the environment. During this period, animals have controlled access to food and free access to water with a temperature of 20 ± 5°C in a 12–12 h light/dark cycle.

#### 2.1.2. Ethical Approval

This research work was approved by the Noakhali Science and Technology University (NSTU) Ethical Review Committee (approval no. NSTU/SCI/EC/2022/117).

#### 2.1.3. Treatment Schedule

At first, animals were divided into two groups (shaped and nonshaped groups, *n* = 8) to judge the necessity of shaping. Later, for the main experiment (single pellet reaching task), the mice were randomly divided into two groups (*n* = 8) as follows:

  Group 1: Control group—given normal saline (1 ml/kg b.w. of mice, orally) for seven days. Mice were given the saline solution by mice feeding needle.
  Group 2: The treatment group was given NSO (1 ml/kg b.w. of mice orally) for seven days. Based on the body weight of the mouse, we have calculated the volume of NSO needed to administered, and then the volume was adjusted up to 0.5 ml by adding normal saline (0.9% NaCl solution) and 1% Tween 80. Later, NSO was administered orally by mice feeding needle during the treatment period. Finally, we also checked the locomotion and anxiety behavior of the mice during the experimental period by using an open field maze. Mice were subdivided into two groups (control and NSO-given groups, *n* = 8).

#### 2.1.4. Acquisition of Research Materials

Normal saline (NaCl 0.9%) was collected from Opsonin Pharma. High-quality *Nigella sativa* oil was purchased from the local pharmacy, and the oil was stored in the laboratory until use.

#### 2.1.5. Behavioral Tasks (Open Field Maze Test)

Before starting the motor behavioral test (single pellet reaching task), animals are tested to assess their anxiety. For this reason, we have performed the open field maze test, which is extensively used to evaluate rodent's locomotion and anxiety behavior [25]. For mice, the test area typically consists of a (42 cm × 42 cm × 42 cm) PVC-box, and a camera is used to monitor the movement into and around the central and peripheral regions of the box. The maze floor was cleaned after testing each mouse using 70% ethanol. Stressed mice show less activity in the open field and increased stereotypical behavior. In addition, they prefer staying close to the walls and traveling more in the periphery. These signs of the mice are considered anxiety-like behavior. On the other hand, mice with lower anxiety tend to spend more time in the central, open area of the box. Mice were not habituated to the box before the test. An individual mouse was placed in the middle of the test box, and its movements were recorded for 5 minutes. The total movements, distance traveled, etc. are recorded by a video camera and registered in the computer.

#### 2.1.6. Single Pellet Reaching Task

To assess the effect of NSO on motor skill learning of mice, we used a single pellet reaching task (SPRT) as described previously [26]. The task has two periods, shaping and training. Mice were food restricted to keep approximately 90% body weight of the original weight during the whole process. Two mice from the same cage were placed in the chamber for 20 minutes on shaping day one to adapt to the environment; on shaping day two, a single mouse was placed in the chamber for 20 minutes. For shaping, ten food pellets were fed daily to each mouse to teach them to take food pellets. On the third day of shaping, a feeding dish with food pellets was positioned in front of the centre slit. The food reward can be obtained by the mouse by having it pass through the slit with either hand. The experiment ended when each mouse had completed 20 instances of reaching efforts. The hand that displays a preference of more than 70% is the dominant hand. Mice made 40 tries in 20 minutes daily to get food pellets through the slit with the dominant hand during the training session. Only efforts made with the dominant hand were counted. The test apparatus was made of an acrylic box (19.5 cm × 8 cm × 20 cm), presenting a 1 cm wide vertical slit running up in front of the box. A 0.2 cm thick plastic shelf (8.3 cm  long and 3.8 cm wide) was mounted, which was 1.1 cm from the floor on the box. Mice received the restricted diet (5 g/day/animal). Ten millet seeds (as food pellets) were given once at a time and were placed in the indentation, spaced 1 cm away from the slit and centred on its edges. Success was scored when animals could put the food pellet in their mouth on the first attempt without dropping. If an animal reached through the slit and obtained a food pellet, the reach was scored as a success. If an animal knocked the food away or dropped the food after grasping it, the reach was scored as a miss.

#### 2.1.7. Statistical Analysis

In this study, all results were expressed as mean ± standard deviation (SD), where *n* = 8. Experimental data found in shaping/nonshaping experiments, SPRT, and OFM were analyzed using two independent sample *t*-tests (parametric approach) and the Mann–Whitney U test (nonparametric approach). *P* < 0.05 was considered statistically significant in all cases. IBM-SPSS statistics 20 software was used to perform the *t*-test and Mann–Whitney *U* tests. The software package GraphPad Prism (5^th^ edition) was used for data analysis and graphical representation.

### 2.2. *In Silico* Procedures

#### 2.2.1. Compound Information Collection and Optimization

The 3D structure of the five best compounds of *N. sativa* oil (NSO) was downloaded from the PubChem database. For better understanding of docking studies, 3D structures of glutamic acid, a parent ligand of the AMPA receptor, were also collected from the PubChem database. After that, all compounds were optimized with DFT, employing Becke's exchange functional, combining Lee, Yang, and Parr's (LYP) correlation functional [27–29]. Due to high accuracy, a 6-31G basis set was used to optimize these compounds. Each compound's electronic energies, enthalpies, Gibbs free energies, and dipole moments were examined. The energies of frontier HOMOs and LUMOs were used to calculate the hardness and softness of all compounds. The following equation estimated the compound's hardness (*η*) and softness (S) using the Parr and Pearson interpretation of DFT [30] and Koopman's theorem [31].

(1) $$\begin{array}{c} \eta =\frac{ℇHOMO-ℇLUMO}{2}\text{,}\\ S=\frac{1}{\eta }\text{.} \end{array}$$

#### 2.2.2. Docking Study of the Molecules

Human iGluR (AMPAR, PDB ID: 3RN8) three-dimensional crystal structure was obtained from the Protein Data Bank. Discovery Studio 2020 client software was used to clean the receptor for molecular docking. The Swiss-PDB viewer was used for energy minimization of the receptor, and the calculations were performed in vacuo with the GROMOS 9643B1 parameter set. To input the protein and ligands for docking analysis, AutoDock Vina was utilized, and AutoDock Tools (ADT) of the PyRx software package was used to convert the structure from pdb to a pdbqt format. For X, Y, and Z, the AutoDock Vina grid box sizes were maintained at 58.81735, 61.2066, and 72.8273. The binding affinity of the ligand was measured in kcal/mol unit, which is basically negative in number (negative score). After the postdocking analysis, the best compounds were judged as AMPA receptor agonists compared to the parent ligand.

#### 2.2.3. ADMET Analysis

Evaluating pharmacokinetic parameters is essential in determining the chemical substance as a drug. *In silico* ADMET analysis can consider and predict the pharmacokinetic properties of the compounds. We have used the SwissADME and pkCSM server for ADMET analysis of the selected compounds described previously. These two servers can forecast essential parameters such as human intestinal absorption, Caco-2 cell permeability, skin permeability, CYP450 metabolism, VDss, blood-brain barrier (BBB) permeation, OCT2 substrate, and toxicity properties. Since SMILE is an input format for a compound in this database, the canonical SMILE format of each compound was obtained from the PubChem database and run for ADMET analysis.

## 3. Results

### 3.1. In Vivo Experimental Results

#### 3.1.1. Effect of Shaping Phase on Motor Skill Learning of the Mice

**Figure 1:**
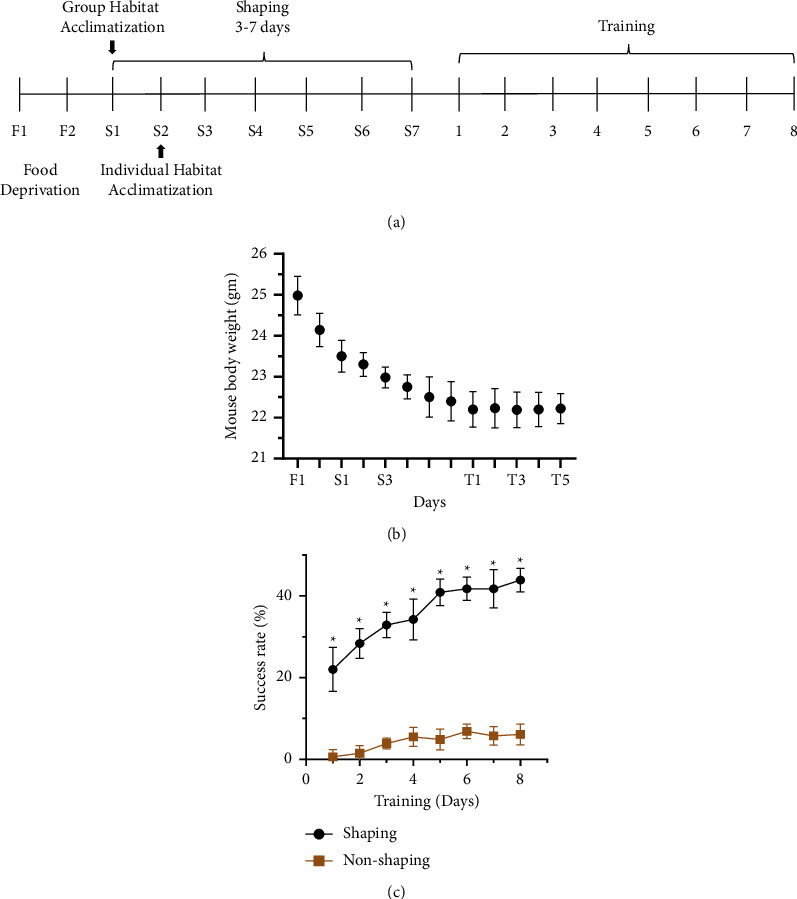
Importance of shaping phase during training of animals. (a) A general timeline of the experimental design. (b) Trend of the body weight of animal during food deprivation which is indicated by F, whereas S = shaping and T = training. (c) Average success rate during training for shaping and nonshaping mice (*n* = 8). Data are represented as mean ± SD, and ^*∗*^ indicates that variation is statistically significant (*P* < 0.05).

This portion of the experiment was carried out to examine how the shaping phase affected the acquisition of motor skills. Here, a total of 16 mice were considered. These mice were divided independently into two equal groups (*n* = 8). Eight consecutive days of training were incorporated to observe the effect of the shaping phase on motor skill learning (Figure 1).

As shown in Figure 1, the mice which were subjected to shaping (shaping group) performed better throughout training than mice which did not get any shaping (nonshaping group). The weights of the mice had not changed much over this time (Figure 1(b)). In comparison to the nonshaping group, the average success rate for each mouse in the shaping group is higher (Figure 1(c)). Student's *t*-test was run to determine the significance of the shaping results [32].  Table S1 has additional information. It was found that the *P* value of the *t*-test statistic is significant at a 5% level of significance (*P* < 0.05), showing that the average success rate was higher in the shaping group than that in the nonshaping group. In addition, the Mann–Whitney *U* test [33] has been done to verify the claim of the significance of the *t*-test. We found that the *P* value of the statistic is 0.001 (*P* < 0.05), which indicates that there is a significant difference in the average success rate for the shaping and nonshaping groups (Table S2). The results of the two tests mentioned above showed a considerable impact of the shaping phase on training.

#### 3.1.2. The Effect of NSO during the Shaping Period Modulates Motor Skill Learning

**Figure 2:**
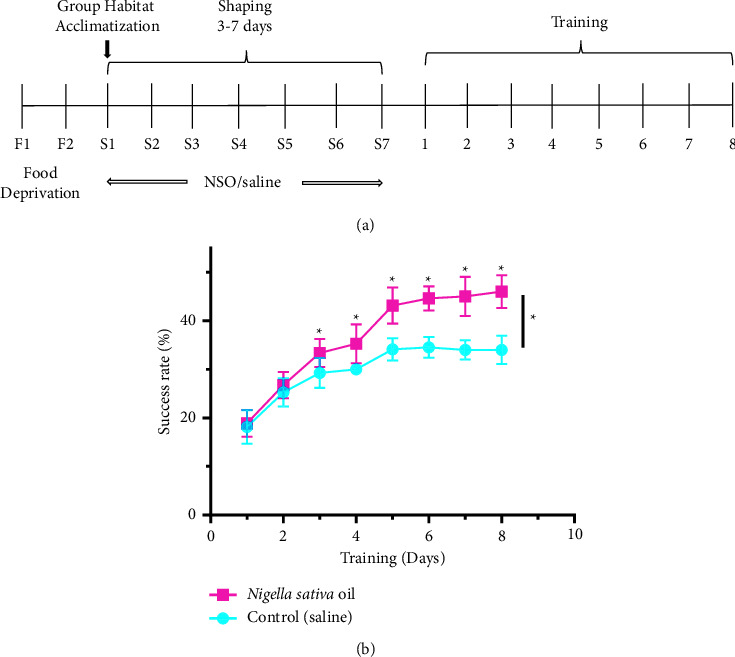
Representative results of NSO ingestion during the shaping phase. (a) A general timeline of the experimental design. (b) Average success rate during training with the treatment of NSO and control (saline) mice (*n* = 8). Data are represented as mean ± SD, and ^*∗*^ indicates that variation is statistically significant (*P* < 0.05).

In this section of the experiment, the mice that were shaped were divided into two groups (*n* = 8 in each group) to see whether NSO significantly influences motor skill learning. One group was given NSO, and another group was given control (saline). Each mouse was observed for eight days, and an arithmetic mean was used to find the average success rate for each mouse. The outcome of the experiment indicated above is shown in Figure 2.

During trial experiments, Day-1 and Day-2, it was discovered that the success rates for the NSO and control groups were nearly identical. An increasing trend of success rate was seen in NSO-treated group beginning from Day-3 to Day-8. On the eighth day of the trial, the disparity reached its peak. This outcome demonstrates a significant impact of NSO on mice behavior. An independent sample *t*-test has been used to determine the significance of this difference (Table S3). Here we also performed the Mann–Whitney *U*test[34] to verify the claim of the significance of the *t*-test [35] (Table S4). It was found that the average success rate due to ingestion of NSO by the trained mice increased significantly compared to the control groups (given saline water).

#### 3.1.3. Assessment of the Anxiety Behaviors before the Motor Skill Learning Test of the Mice

**Figure 3:**
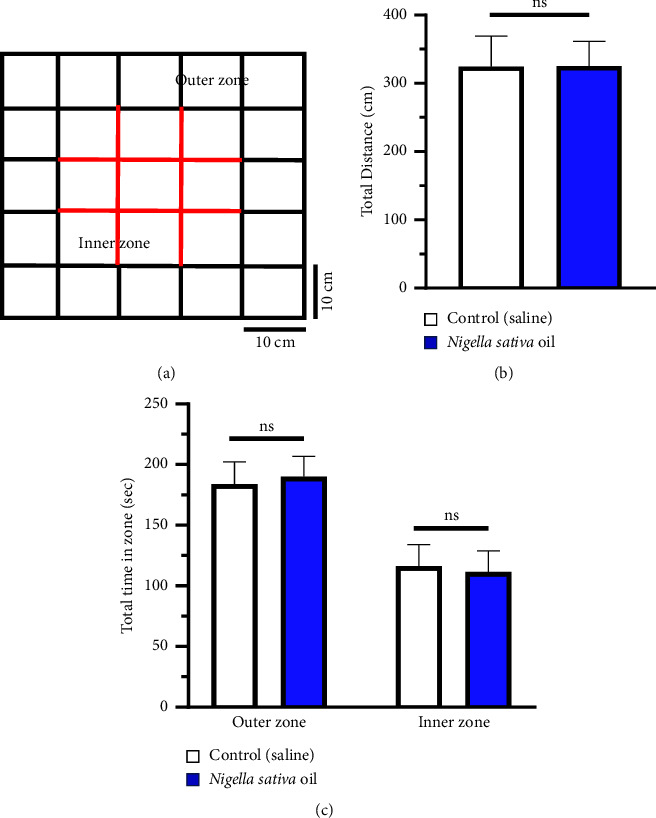
Representation results of open field maze. (a) Zone overlay used to interpret tracking data for thigmotaxis from the OFM. (b) Total distance traveled in the OFM. (c) Time spent in inner and outer zones of the OFM (*n* = 8). Data are represented as mean ± SD, and ns indicates that variation is not statistically significant (*P* > 0.05).

An open field maze (OFM) test was conducted prior to the motor skill learning test to determine whether or not the tested animals experienced anxiety. As shown in Figure 3, there is no significant anxiety-like behavior (*P* > 0.05) found between control and NSO grouped mice (*n* = 8 for each group). To identify any discrepancies, a statistical analysis was performed on the sum of the distance and time spent in the inner and outer zones of the OFM. Significant variations were not seen between control group and NSO-treated group on mice's performance in the OFM (Figures 3(b) and 3(c)).

### 3.2. *In Silico* Study Assessment

#### 3.2.1. Optimization of Compound and Analysis of Frontier Molecular Orbitals

All compounds were subjected to comprehensive geometry optimization using DFT because a molecule's conformational characteristics significantly impact its physical and chemical properties.  Table 1 displays the chemical's stoichiometry, electronic energy, enthalpy, Gibbs free energy, and dipole moment. The structural characteristics of a compound, including its energy, partial charge distribution, and dipole moment, are obviously greatly influenced by changes in its structure. Glutamic acid was found to have the highest energy, enthalpy, and Gibbs free energy, whereas carvacrol displayed the highest dipole moment in nature, measuring 1.438 Debye indicating high polarity.

The frontier orbitals, or major molecular orbitals of a molecule, are assumed to describe the chemical reactivity and kinetic stability of the molecule. Frontier orbitals are those that are present in a molecule and consist of the highest occupied molecular orbital (HOMO) and the lowest unoccupied molecular orbital (LUMO). The values of the two global chemical descriptors, hardness, and softness, which are also determined for all compounds, are shown in Table 2, along with the orbital energies. It was found that thymoquinone has the highest level of softness.

#### 3.2.2. Molecular Docking Analysis

**Figure 4:**
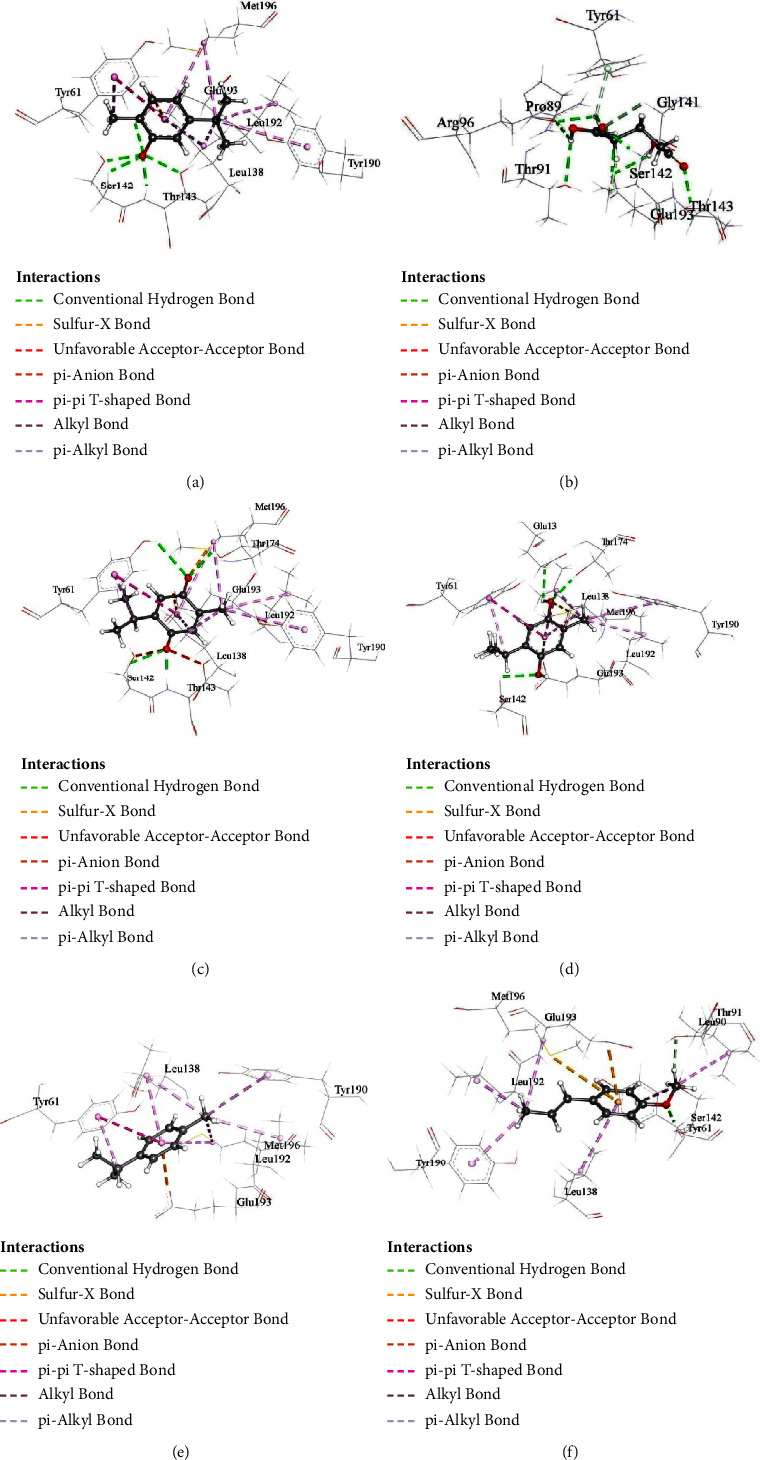
Possible 3D interactions of (a) carvacrol and AMPA-R, (b) glutamic acid and AMPA-R, (c) thymoquinone and AMPA-R, (d) thymohydroquinone and AMPA-R, (e) *p*-cymene and AMPA-R, and (f) *t*-anethole and AMPA-R.

**Figure 5:**
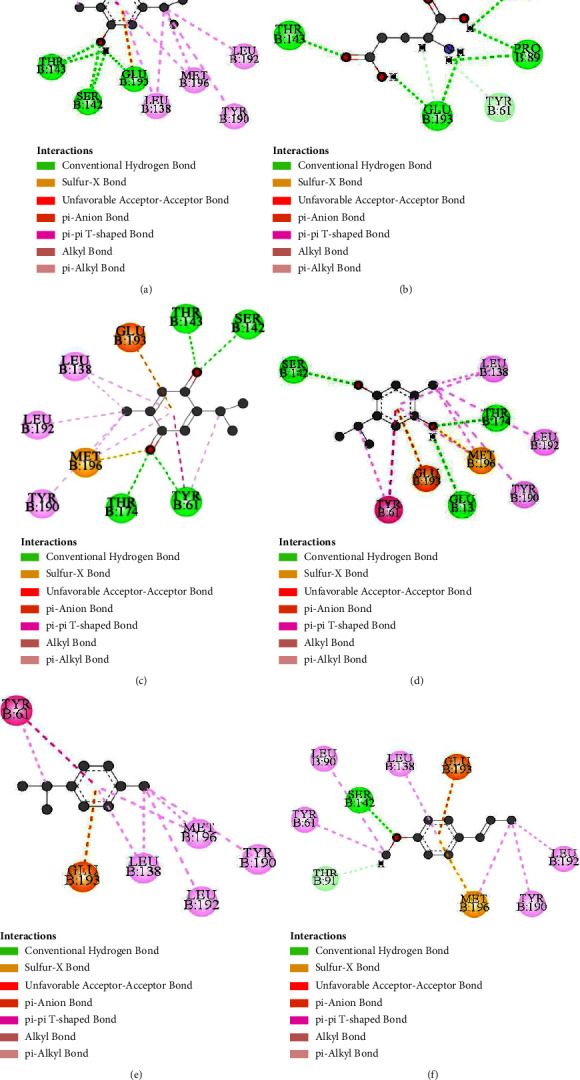
Possible 2D interactions of (a) carvacrol and AMPA-R, (b) glutamic acid and AMPA-R, (c) thymoquinone and AMPA-R, (d) thymohydroquinone and AMPA-R, (e) *p*-cymene and AMPA-R, and (f) *t*-anethole and AMPA-R.

The binding manner of selected substances was examined using molecular docking by AutoDock Vina. Molecular docking, one of the most well-liked methods for structure-based drug design, allows researchers to look at the atomic-level interactions between a small molecule and a protein. All selected compounds are docked into the same binding pockets of AMPA receptor using similar optimized docking conditions. The outcomes of the docking analysis showed that all compounds, along with the parent compound, obtain binding affinities ranging from −5.1 to −6.6 kcal/mol (Tables S5–S7). The binding affinity of thymoquinone and carvacrol was found to be close to that of glutamic acid. Postdocking analysis revealed that glutamic acid formed 11 hydrogen bonds with TYR61, ARG96, PRO89, THR91, ARG96, GLY141, SER142, THR143, and GLU193 residues. Carvacrol formed 5 hydrogen bonds with GLY141, SER142, and GLU193 residues and 8 hydrophobic bonds with TYR61, LEU138, TYR190, LEU192, and MET196. Carvacrol also showed one electrostatic interaction with GLU193 residue. Thymoquinone, the most abundant compound of *N. sativa*, showed four hydrogen bonds with TYR61, SER142, THR143, and THR174 residues. This compound also showed eight hydrophobic and one electrostatic interaction with TYR61, LEU138, TYR190, LEU192, MET196, and GLU193 residues. Compared to the docking result of glutamic acid, the remaining three compounds—thymohydroquinone, *p*-cymene, and *t*-anethole—have lower binding affinity and fewer bonds with active amino acids. On the other hand, thymoquinone formed hydrogen bond-like glutamic acid with TYR61, SER142, and SER143 residues. Carvacrol also binds with four amino acid residues of the AMPA receptor, which is important for glutamic acid binding. Possible 3D and 2D interactions of carvacrol, glutamic acid, thymoquinone, thymohydroquinone, *p*-cymene, and *t*-anethole with AMPA receptor are shown in Figures 4 and 5.

#### 3.2.3. Prediction of ADMET Profile

This method makes it simple to ascertain the pharmacokinetic profile of any substance because computation is affordable and time-efficient and produces results that are near to experimental data. To determine whether our selected compounds could act as drugs, we used the pkCSM and SwissADME servers to estimate their ADMET profile. To forecast how well oral medications would be absorbed, the human epithelial Caco-2 monolayer of cells is frequently utilized as *in vitro* model of the human intestine. The highest Caco-2 permeability is >0.90. ADMET analysis results of our targeted compounds showed that all the compounds have high Caco-2 permeability, as shown in Table 3. The majority of medications taken orally are absorbed in the human gut. These compounds show high intestinal absorption in humans (>90%).

The bioavailability score indicates that all the compounds are highly bioavailable. On the other hand, skin permeability is an essential factor for considering and preparing transdermal drug delivery systems. Thymohydroquinone exhibited low skin permeability, whereas the other four compounds had high skin permeability (low skin permeability value is <−2.5).

The compound's capacity to enter the brain region is an important parameter for the development of the neuroprotective drug. It was found that all the selected compounds can cross the BBB (logBB> 0.3, which is considered to cross the BBB easily) (Table 3). CYP450 is an essential enzyme for drug metabolism. Only one major isoform, CYP1A2, is affected by all compounds except thymoquinone. pkCSM hypothesizes that not all the selected substances are OCT2 substrates. Toxicity test results suggested that all five ligands are safe and nontoxic.

## 4. Discussion

One remarkable aspect of the brain is its ability to alter neuronal activity and bring about long-lasting changes in neural circuits in response to new learning experiences. This study aimed to examine the effect of *N. sativa* oil (NSO) on motor learning skills using a standard behavioral model, a single pellet reaching task in mice. NSO contains various compounds that affect the different body parts to different extents. Therefore, the exact mechanism of the positive modulating effect of NSO is challenging to explain which could have been done easily in the case of a study with one compound.

Figure 1 illustrates the significance of the shaping step in training mice to successfully grasp food pellets more often than untrained mice. In this investigation, we discovered that NSO considerably enhances the trained animal's ability to master motor skills. It was found in Figure 2 that from the beginning of the experiment, 1st and 2nd day, success rates were almost the same for the NSO and control groups. However, an increasing order trend was observed from the 3rd day for the NSO-treated group compared to the control, and this difference was statistically significant (*P* < 0.05). The tested mice had not experienced any anxiety that would have affected the outcomes of the motor learning tests conducted before and during the main trials (Figure 3). Previous investigation reported that NSO possesses anti-inflammatory, antioxidant, anticholinesterase, and antimicrobial properties [17]. It is well known that inflammatory processes are one of the significant factors that may contribute to CNS injury, including deficits in complex motor skills [36]. A previous study revealed that inflammation decreased dopamine and dopamine metabolites in cerebrospinal fluid, which correlated with reduced motor function [35]. NSO might play a role in this process or revert it, but it requires further study for confirmation.

Previous studies reported that increased oxidative stress in the brain has been associated with motor deficits [34]. Since oxidative stress is characterized by an imbalance in radical production of reactive oxygen species (ROS) and antioxidative defense, both are believed to have a significant role in the process of neurodegeneration and motor deficit [37], and therefore, substances like NSO, which have antioxidant properties, might improve motor learning skills (Figure 2). Moreover, thymoquinone is one of the main constituents present in NSO which is reported for its antioxidant activities. Although fractions rich in thymoquinone were found to be most potent in terms of antioxidant capacity, previous research indicates that the protective effects of *N. sativa* may be due to thymoquinone and other antioxidants [38].

The cerebellum plays a crucial role in motor coordination [39]. NSO has been described previously for the protective and ameliorative effects on the prefrontal and frontal cortical pyramidal neurons, dentate gyrus large granular cells, and hippocampal CA pyramidal neurons, thereby enhancing motor skill learning [40]. NSO is also reported to improve the neurogenic cells in the hippocampus, hence contributing to enhancing memory. It can rebuild neuronal integrity and functions [21]. However, it needs more investigation to elucidate the molecular mechanism of the site of action of NSO.

In silico study analysis reported that carvacrol showed the highest dipole moment representing high polarity in nature (Table 1). It is well known that the higher the dipole momentum, the higher the solubility rate. Thymoquinone displayed the lowest HOMO-LUMO gap and hardness (Table 2), demonstrating that the molecule is more reactive than other compounds [31]. The binding affinity of our selected compounds with AMPA receptors was observed using molecular docking by AutoDock Vina. Carvacrol was found to form hydrogen bonds with AMPA receptor sites GLY141, SER142, and GLU193 and hydrophobic bonds with TYR61, which is similar to the binding trend of glutamic acid. On the other hand, thymoquinone showed four hydrogen bonds with TYR61, SER142, THR143, and THR174 residues, which are also similar to glutamic acid. The other three compounds have lower binding affinity and fewer bonds with active amino acids. The formation of hydrogen bonds between targeted compounds and receptors can effectively modulate the activity of enzymes and show the potent pharmacological response [41]. ADMET analysis reported that all the compounds have high intestinal absorption in humans (>90%) and high skin permeability except thymohydroquinone and can cross the BBB easily (logBB> 0.3) (Table 3). Toxicity test results suggested that all five ligands are safe and nontoxic. Based on the above reasons, it can be said that both thymoquinone and carvacrol may serve as promising candidates for AMPA receptor agonists.

Skillful voluntary motions need the primary motor cortex (M1). Layers 2/3 and 5 (L2/3 and L5) of the M1 cortex, which is reorganized during learning, are necessary for developing motor skills [42]. Investigations of the virus-mediated in vivo gene delivery with in vitro patch-clamp recordings reported that AMPA receptor delivery into the synapses is involved in synaptic strengthening [43]. LTP-like plasticity of M1 contributes to motor skill retention, which could correlate with the previous study that an increase of postsynaptic AMPA receptors guides synaptic strengthening among layer 2/3 neurons [6]. According to the in silico study analysis, thymoquinone and carvacrol present in NSO may serve as AMPA receptor agonists. In addition, it might play a role in synaptic strengthening (LTP) in layer 2/3 neurons of the primary motor cortex and hence promote experience-dependent motor skill learning. Further in vivo and in vitro electrophysiology and imaging studies of thymoquinone and carvacrol on AMPA receptors were required for confirmation.

## 5. Conclusion

In this investigation, we studied the effect of *N. sativa* oil on motor skill learning modulation in mice models. Due to the ingestion of NSO, the average success rate of the mice in single pellet reaching tasks increased significantly compared to the control groups, which resembles the improvement of motor learning. Molecular docking and ADMET analysis modeling predicted that thymoquinone and carvacrol meet most of the criteria of an ideal ligand. Thymoquinone is much more reactive among the compounds found in NSO. Based on the experimental data and *in silico* studies, we have concluded and predicted that the motor skill improvement effect found in the NSO may be due to the presence of thymoquinone and carvacrol antioxidants. However, further research should be done to find the exact compound and mechanism of this motor learning improvement.

## Figures and Tables

**Table 1:** The stoichiometry, electronic energy, enthalpy, Gibbs free energy, and dipole moment (Debye) of thymoquinone, thymohydroquinone, *p*-cymene, glutamic acid, carvacrol, and *t*-anethole.

Name	Stoichiometry	Electronic energy	Enthalpy	Gibbs free energy	Dipole moment (Debye)
Thymoquinone	C_10_H_12_O_2_	−538.53	−538.53	−538.58	0.182
Thymohydroquinone	C_10_H_14_O_2_	−539.73	−539.73	−539.78	0.149
*p*-Cymene	C_10_H_14_	−389.30	−389.31	−389.36	0.086
Glutamic acid	C_5_H_9_NO_4_	−551.48	−551.48	−551.58	1.289
Carvacrol	C_10_H_14_O	−464.52	−464.52	−464.58	1.438
*t*-Anethole	C_10_H_12_O	−463.30	−463.30	−463.35	1.179

**Table 2:** Energy (atomic unit) of HOMO, LUMO, gap, hardness, and softness of the compounds.

Molecules	*ε*_HOMO_	*ε*_LUMO_	Gap	*η* (hardness, gap/2)	S (softness, 1/hardness)
Thymoquinone	−0.259	−0.118	0.140	0.070	14.192
Thymohydroquinone	−0.190	0.006	0.197	0.099	10.152
*p*-Cymene	−0.226	0.005	0.232	0.116	8.613
Glutamic acid	−0.245	−0.001	0.244	0.122	8.197
Carvacrol	−0.211	0.007	0.218	0.109	9.176
*t*-Anethole	−0.195	−0.015	0.210	0.105	9.487

**Table 3:** Pharmacokinetic profile and toxicity prediction of the selected compounds.

Parameter		Thymoquinone	Carvacrol	Thymohydroquinone	*p*-Cymene	*t*-Anethole
Absorption	Water solubility (log mol/L)	−1.594	−2.043	−2.007	−4.081	−2.936
	Caco-2 permeability (log Papp, cm/s)	1.344	1.52	1.066	1.527	1.669
	HIA (%)	98.531	91.806	91.659	93.544	95.592
	Bios (from SwissADME) (bioavailabilty score)	0.55	0.55	0.55	0.55	0.55
	Skin permeability (logKp cm/s)	−2.533	−1.644	−2.949	−1.192	−1.139

Distribution	VDss (human) (logL/kg)	−0.034	0.451	0.33	0.697	0.343
	BBB permeability (logBB)	0.372	0.383	0.339	0.478	0.529
	BBB permeability (SwissADME)	Yes	Yes	Yes	Yes	Yes

Metabolism	CYP2D6 substrate	No	No	No	No	No
	CYP3A4 substrate	No	No	No	No	No
	CYP1A2 inhibitor	No	Yes	Yes	Yes	Yes
	CYP2C19 inhibitor	No	No	No	No	No
	CYP 2C9 inhibitor	No	No	No	No	No
	CYP 2D6 inhibitor	No	No	No	No	No
	CYP 3A4 inhibitor	No	No	No	No	No

Excretion	Total clearance	0.225	0.207	0.28	0.239	0.268
	Renal OCT2 substrate	No	No	No	No	No

Toxicity assays	Ames toxicity	No	No	No	No	No
	Oral rat acute toxicity (LD_50_, in mol/kg)	1.68	2.251	2.201	1.827	1.798

## Data Availability

The data used to support the findings of this study are available on request from the corresponding authors. Supplementary data (including Tables S1–S7) are included within the supplementary information file.

## Abbreviations

NSO: *Nigella sativa* oil
M1: Motor cortex
LTP: Long-term potentiation
AMPA: *α*-Amino-3-hydroxy-5-methyl-4-isoxazolepropionic acid
CE: Cerebellar norepinephrine
CNS: Central nervous system
SPRT: Single pellet reaching task
OFM: Open field maze
HOMO: Highest occupied molecular orbital
LUMO: Lowest unoccupied molecular orbital.

## Supplementary Materials

Supplementary Materials Table S1: Levene's test for equality of variances. Table S2: independent samples Mann–Whitney *U* Test. Table S3: Levene's test for equality of variances. Table S4: results of the Mann–Whitney *U* test for the eight consecutive days. Table S5: molecular docking analysis of glutamic acid and thymoquinone with AMPA receptor. Table S6: molecular docking analysis of *p*-cymene and *t*-anethole with AMPA receptor. Table S7: molecular docking analysis of carvacrol and thymohydroquinone with AMPA receptor.
