# Supplementary material for: Nigella sativa Oil Improves Motor Skill Learning of Albino Mice: In Vivo and In Silico Investigations
Source: Evid Based Complement Alternat Med. 2023 Aug 25;2023:8498066. doi: 10.1155/2023/8498066 (PMC10473895; doi:10.1155/2023/8498066)

**SUPPLEMENTARY DATA**

Nigella sativa**oil improves motor skill learning of albino mice:**In vivo**and**in silico**investigations**

Md. Siam Hossain^1^, Abu Baker Seddique^1^, Suraiya Sharmin^2^, Md. Mamun Or Rashid^1^, Arifin Islam^3^, Md. Monir Hossain^4^

**RESULTS**

**Effect of shaping phase on motor skill learning**

Two independent sample t-test and has been applied to test whether the mean success rate of the shaping and non-shaping group are equal or not. The hypothesis for two independent sample t-test is -

Ho: Average success rate in the two groups are equal.

H_1_: Average success rate is greater in the shaping group then those in the non-shaping group.

The result of the t-test has been given in the following **Table S1**:

|  | Levene's Test for Equality of Variances | | t | DF | *P*value |
| --- | --- | --- | --- | --- | --- |
| Equal Variance not Assumed | F | *P*value |  |  |  |
|  | 5.041 | .041 | -12.043 | 14 | .000 |

From the Table S1, Levene's test for equality of variances shows that equal variance was not assumed in the t-test (*P*< 0.05) and *P* value of the t test statistic is significant at 5% level of significance (*P*<0.05) indicated that average success rate was greater in the shaping group then those in the non-shaping group.

Also the Mann-Whitney U test (1947) has been used to test whether the distribution of the success rate for the training in shaping and non-shaping group is equal or not. The test has been incorporated, because there may be violation of the distributional assumption need to apply for the parametric test. (Hodges JL, Lehmann EL (1956). This test also verifies the result of the t-test. In this test, null hypothesis and alternative hypothesis was-

H_0_: There is no significant difference in the distribution of success rate for the shaping and

non-shaping group.

H_1_: There is significant difference in the distribution of success rate for shaping and

non-shaping group.

The result of the test has been given in the **Table S2:**

| Null hypothesis | Test | *P*value of the test statistic | Decision |
| --- | --- | --- | --- |
| There is no significant difference in the distribution of success rate for the shaping and non-shaping period. | Independent samples Mann- Whitney U Test | .001 | Reject the null hypothesis |

From the above tables (Table S1, and Table S2), we have seen that the *P* value of the statistic is .001 (*P*< 0.05), which indicates that there is a significant difference in the average success rate for the shaping and non-shaping group.

The result of the above two tests clearly indicated that shaping period has significant effect on training.

**Effect of *Nigella sativa* oil (NSO) during the shaping period modulates motor skill learning:**

Independent sample t-test has been performed to assess the experimental results. The null and alternative hypothesis for the t-test was -

H_0_: There is no significant difference in the average success rate between the two groups.

Against

H_1_: The average success rate is higher in the in the NSO group.

The result of the test has been given in **Table S3**:

| **Day** |  | **Levene's Test for Equality of Variances** | | **t** | **DF** | *P***value** |
| --- | --- | --- | --- | --- | --- | --- |
|  |  | F | *P*value |  |  |  |
| 1 | Equal variance assumed | .317 | .582 | -.478 | 14 | .640 |
| 2 | Equal variance assumed | .501 | .491 | -1.065 | 14 | .305 |
| 3 | Equal variance assumed | .049 | .828 | -2.779 | 14 | .015* |
| 4 | Equal variance not assumed | 5.523 | .034 | -3.594 | 7.99 | .007* |
| 5 | Equal variance assumed | 3.234 | .094 | -5.824 | 14 | .000* |
| 6 | Equal variance assumed | .024 | .880 | -8.698 | 14 | .000* |
| 7 | Equal variance assumed | 4.342 | .056 | -6.908 | 14 | .000* |
| 8 | Equal variance assumed | .241 | .631 | -7.589 | 14 | .000* |

* Indicates significance at 5% level of significance.

From the Table S3, we have observed that Levene'stest for equality of variances is significant at 5% level of significance (*P*> 0.05) for the fourthday only, which indicated that only for fourth day equal variance has not been assumed for the t-test and for rest of the day equal variance has been used for test for the two groups. Further from the t-test we have observed that *P*value of the t-test is significant at 5% level of significant (*P*< 0.05) from the third day of the experiment, indicating that the null hypothesis of the equality of two means has be rejected from the third to eighth day which resembles the results that we have found in Figure 2C. To cross check the independent sample t-test results, non-parametric test Mann-Whitney U test has been incorporated.

| **Day** | **Null hypothesis** | **Test** | ***P* value of the test statistic** | **Decision** |
| --- | --- | --- | --- | --- |
| 1 | The distribution of the Success rate among NSO and Control group are same | Independent samples Mann- Whitney U Test | .505 | Accept the null hypothesis |
| 2 | The distribution of the Success rate among NSO and Control group are same |  | .442 | Accept the null hypothesis |
| 3 | The distribution of the Success rate among NSO and Control group are same |  | .015* | Reject the null hypothesis |
| 4 | The distribution of the Success rate among NSO and Control group are same |  | .010* | Reject the null hypothesis |
| 5 | The distribution of the Success rate among NSO and Control group are same |  | .000* | Reject the null hypothesis |
| 6 | The distribution of the Success rate among NSO and Control group are same |  | .000* | Reject the null hypothesis |
| 7 | The distribution of the Success rate among NSO and Control group are same |  | .000* | Reject the null hypothesis |
| 8 | The distribution of the Success rate among NSO and Control group are same |  | .000* | Reject the null hypothesis |

**Table S4:** Results of the Mann Whitney U test for the eight consecutive days.

* Indicates significance at 5% level of significance.

From the Table S4, it can be said that the null hypothesis for the Mann-Whitney U Test is rejected at 5% level of significance (*P*< 0.05) from third day to eighth day indicating that there has been significant difference in the success rate in the NSO group and control group. The result of the Mann-Whitney U test was similar to test result found in t-test at Table S3.

**Table S5**. Molecular docking analysis of glutamic acid and thymoquinone with AMPA receptor.


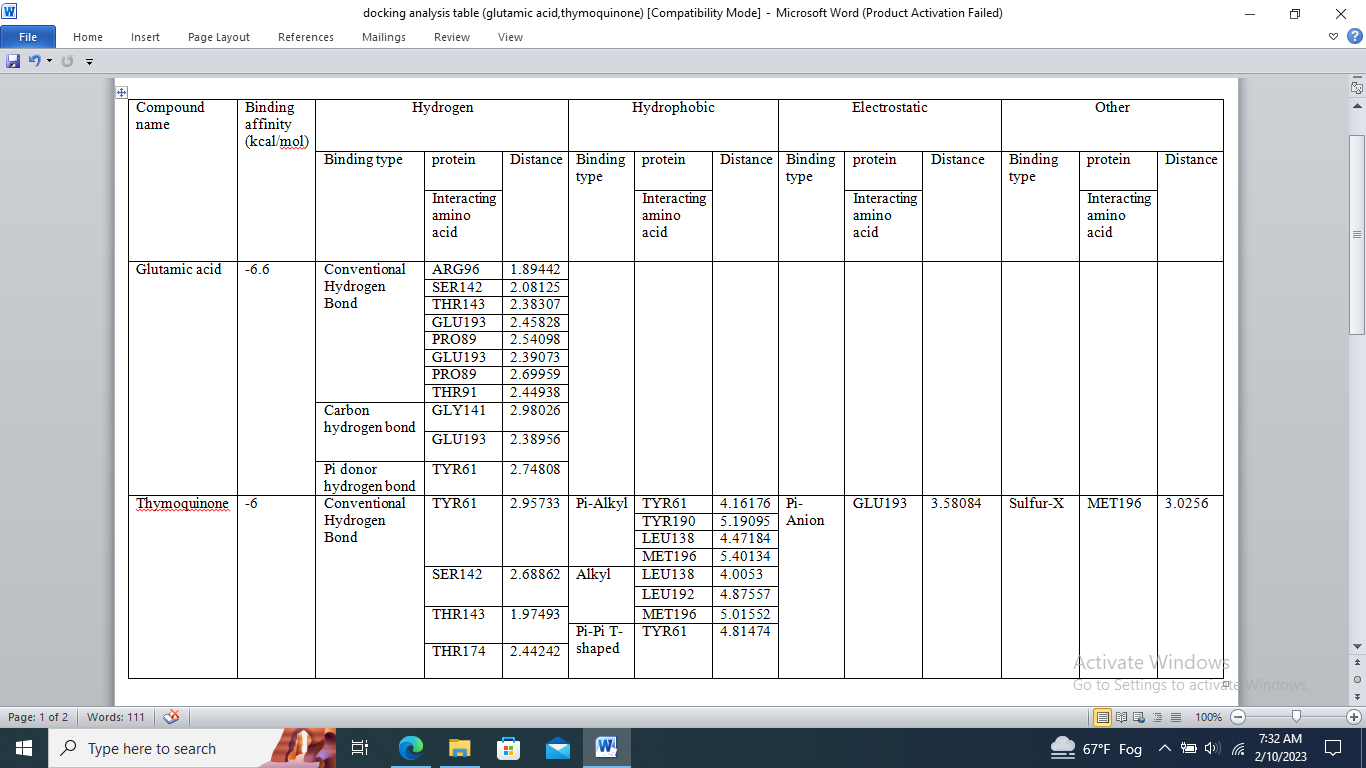


**Table S6**. Molecular docking analysis of *p*-cymene and *t*-anethole with AMPA receptor.


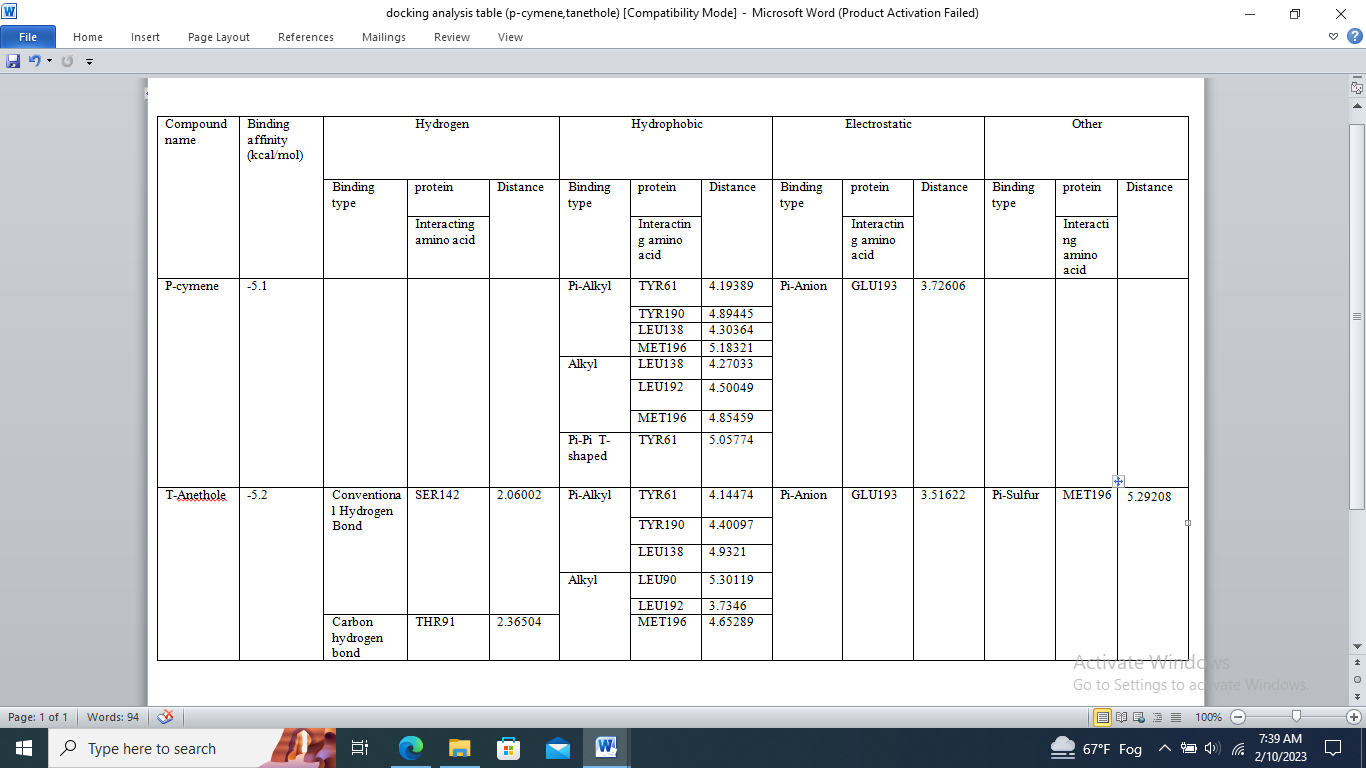


**Table S7**. Molecular docking analysis of carvacrol and thymohydroquinone with AMPA receptor.


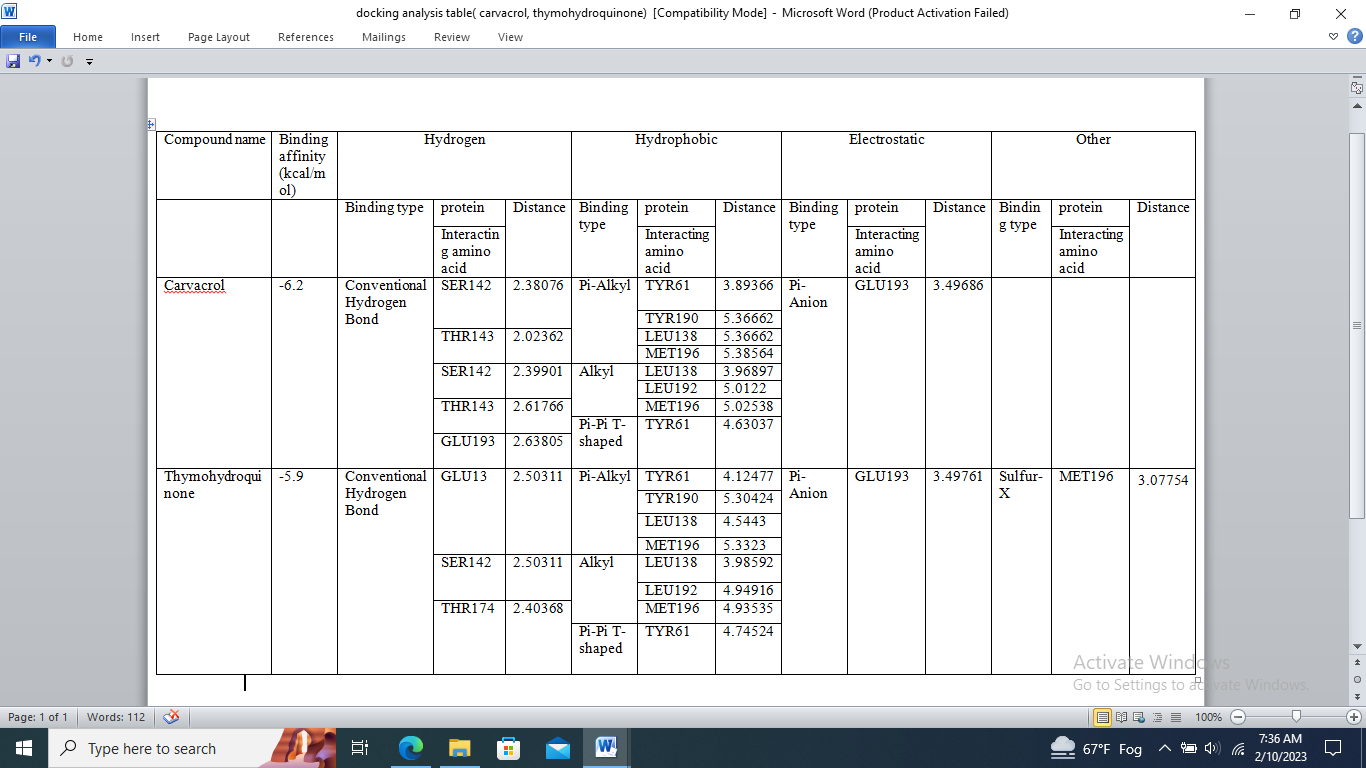

Supplement: Supplementary Materials — Table S1: Levene's test for equality of variances. Table S2: independent samples Mann–Whitney U Test. Table S3: Levene's test for equality of variances. Table S4: results of the Mann–Whitney U test for the eight consecutive days. Table S5: molecular docking analysis of glutamic acid and thymoquinone with AMPA receptor. Table S6: molecular docking analysis of p-cymene and t-anethole with AMPA receptor. Table S7: molecular docking analysis of carvacrol and thymohydroquinone with AMPA receptor. [file 8498066.f1.docx]
